# More intramedullary nails and arthroplasties for treatment of hip fractures in Sweden: Registry analysis of 144,607 patients, 1998–2007

**DOI:** 10.3109/17453674.2010.506631

**Published:** 2010-10-08

**Authors:** Cecilia Rogmark, Curt-Lennart Spetz, Göran Garellick

**Affiliations:** ^1^Department of Orthopaedics, Lund University, Skane University Hospital, Malmö; ^2^Centre for Epidemiology, Swedish National Board of Health and Welfare, Stockholm; ^3^Swedish Hip Arthroplasty Register, Göteborg, Sweden

## Abstract

**Background and purpose:**

The surgical methods for treatment of femoral neck fractures and trochanteric hip fractures vary. We describe the changes in Sweden over the period 1998–2007 and the regional differences in treatment.

**Patients and methods:**

Data on 144,607 patients were drawn from the National Patient Register.

**Results:**

The proportion of femoral neck fractures treated with arthroplasty increased from 10% in 1998 to 52% in 2007. The use of intramedullary (IM) nails for pertrochanteric fractures increased from 5% to 20%, at the expense of the use of different sliding hip screws. In subtrochanteric fractures, the use of IM nails increased from 32% to 72%. Re-admissions within 180 days due to hip complications were more common after internal fixation for femoral neck fractures than after arthroplasty, and more common after intramedullary nailing of pertrochanteric fractures than after use of sliding hip screws. Treatment varied substantially within Sweden, particularly regarding the use of IM nails.

**Interpretation:**

An increase in arthroplasties reflects an evidence-based treatment rationale for femoral neck fractures, whereas the increase in use of IM nails in pertrochanteric fractures lacks scientific support. The geographic variations call for national treatment guidelines. Further clinical trials are needed to solve the treatment issues regarding per- and subtrochanteric fractures.

Intramedullary nails may be taking an increasing share in the treatment of extracapsular fractures in the USA (Anglen and Weinstein 2008). There are few data regarding other countries. The method is promoted at trauma meetings but the scientific basis is not yet convincing (Parker and Handoll 2008). For femoral neck fractures, though, an evidence-based algorithm has been formed (Parker and Gurusamy 2006), with increased use of arthroplasties—at least in the elderly.

During the past decade, swift changes in the treatment of hip fractures have taken place in Sweden. We describe the trends in the use of treatment methods for hip fractures in Sweden during the period 1998–2007, including regional differences and re-admissions.

## Material and methods

Data were extracted from the National Patient Register. The analyses were based on the first period of hospital admission due to acute hip fracture for 144,607 individuals treated between 1998 and 2007 in Sweden. Subgroup analyses were done as described below in Results.

The diagnoses were defined by using the ICD 10-system (WHO 2007) and the surgical procedures were classified by the Swedish version of the NOMESCO Classification of Surgical Procedures (NCSP) (NOMESCO 2008). Femoral neck fractures are classified as S72.0, pertrochanteric fractures as S72.1 and subtrochanteric as S72.2. We used the NFB group of codes to define arthroplasty procedures. Internal fixation for femoral neck fractures was defined as NFJ49 (Hansson hook pins), NFJ79 (Uppsala screws and similar), and NFJ89 (sliding hip screw). Intramedullary nails were searched as NFJ59, whereas extramedullary hip screw and plate was defined as both NFJ69 and NFJ89. Hip complications were defined as M24.3, M24.4 (dislocation), M84.0, M84.1, M84.2 (malunion/nonunion), M87.2 (posttraumatic osteonecrosis), M96.6 (periprosthetic fracture), M96.8, M96.9, (other/unspecified postprocedural musculoskeletal disorders), T81 (complications of procedures), T84 (complications of internal orthopedic prosthetic implants), and T93.1 (sequelae of fractures of the femur).

Information to the National Patient Register is delivered once a year to the Centre for Epidemiology, Swedish National Board of Health and Welfare (EpC) from the 21 county councils in Sweden, as a disc with one data file for the whole county. Every discharge during one year corresponds to one record in that file. Statistical analysis was done with the chi-squared test.

## Results

**Figure 1.**
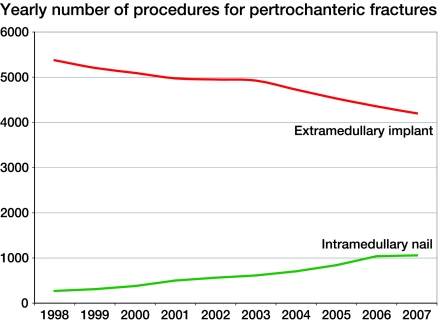
Surgical methods used for pertrochanteric fracture (S72.1) over time.

For pertrochanteric fractures, the number of intramedullary nails increased from 271 in 1998 to 1,059 in 2007 (i.e. from 5% to 20%), at the expense of the use of different sliding hip screws or other extramedullary implants. The latter decreased from 5,374 to 4,200 (from 95% to 80%) (Figure 1). There were no sex or age differences for this fracture type.

**Figure 2.**
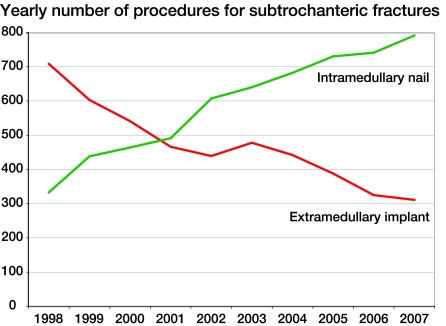
Surgical methods used for subtrochanteric fracture (S72.2) over time.

The increase in the use of intramedullary nails was more prominent for subtrochanteric fractures. They increased from 333 to 791 (32% to 72%), whereas the extramedullary implants correspondingly decreased from 708 to 311 (68% to 28%) (Figure 2). There was no gender difference, but during the 10-year period patients under 70 years got more intramedullary nails than those over 80 (59% and 55%; p = 0.007).

**Figure 3.**
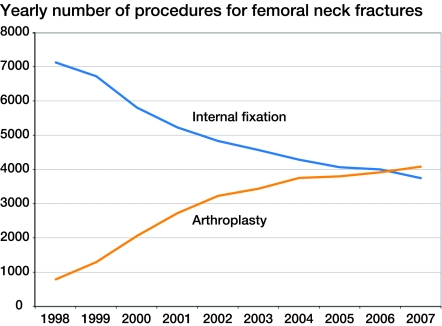
Surgical methods used for femoral neck fracture (S72.0) over time.

The surgical treatment for femoral neck fractures showed an evident change (Figure 3). In 1998, 784 operations (10%) classified as arthroplasties were performed. 7,121 internal fixations were performed (90%). 9 years later, the majority were treated with arthroplasty, 4,078 (52%), in comparison to 3,741 (48%) internal fixations.

**Figure 4.**
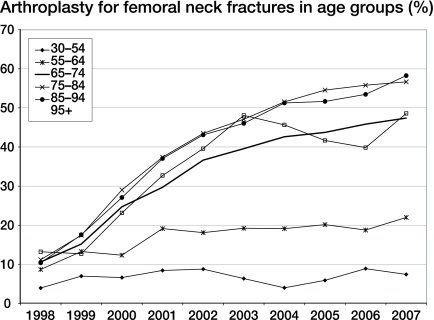
Use of arthroplasty for femoral neck fracture (S72.0) in different age groups over time.

Women were treated with arthroplasty to a greater extent than men (40% and 30%, respectively; p < 0.001). The increase in arthroplasty was most pronounced in patients over 65 years, but an increase was also seen for those between 55 and 64 years (Figure 4).

Regarding the need for re-admission, 33,181 patients with dismissal from hospital after acute hip fracture treatment from January 1, 2005 through June 30, 2007 were analyzed. Patients who died during the hospital stay or who were transferred to another hospital department were excluded before the analysis.

Re-admission to any hospital department within 180 days, regardless of diagnosis, was required for 9,485 patients (29%). The frequency of re-admission was higher after femoral neck fractures than after extracapsular fractures (p = 0.02). Femoral neck fracture patients treated with internal fixation had more re-admissions than those treated with arthroplasty (Table).

Re-admission within 180 days due to hip complications occurred statistically significantly more often after internal fixation for femoral neck fractures than after arthroplasty, and more often after intramedullary nailing of pertrochanteric fractures than after use of a sliding hip screw. Internal fixation for femoral neck fractures showed the highest overall hip-related re-admission rate (9.3%) and sliding hip screw for pertrochanteric fractures showed the lowest (3.8%) (Table 1).

**Table:** Hospital re-admissions within 180 days, 2005–2007

A	B	C	D	E	F	G	H	I	J
S72.00	18,196	Arthroplasty	8,800	2,466	28	0.001	632	7.2	< 0.001
		Internal fixation	9,396	2,835	30		877	9.3	
S72.10	12,470	Intramedullary nail	6,041	1,701	28	0.639	305	5.1	< 0.001
		SHS	6,429	1,786	28		246	3.8	
S72.20	2,515	Intramedullary nail	2,040	563	28	0.788	129	6.3	0.86
		SHS	475	134	28		29	6.1	

A Diagnosis B Alive at discharge from hospital C Method SHS: sliding hip screw, including Medoff plate (biaxial sliding plate). D Alive at discharge from hospital E Re-admitted within 180 days F % G p-value H Re-admission due to hip complication I % J p-value

**Figure 5.**
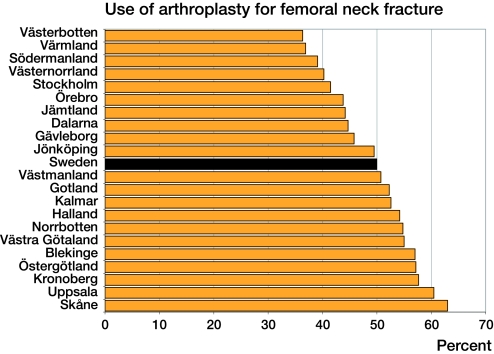
Use of arthroplasty for femoral neck fracture (S72.0) in different Swedish counties, 2005–2007

**Figure 6.**
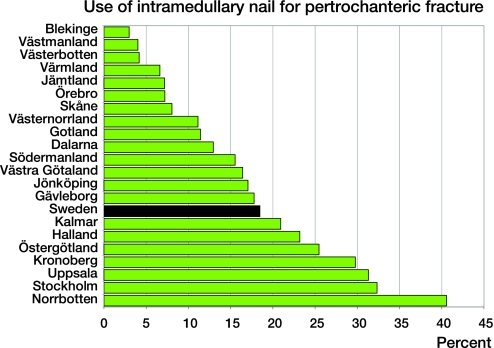
Use of intramedullary nail for pertrochanteric fracture (S72.1) in different Swedish counties, 2005–2007

**Figure 7.**
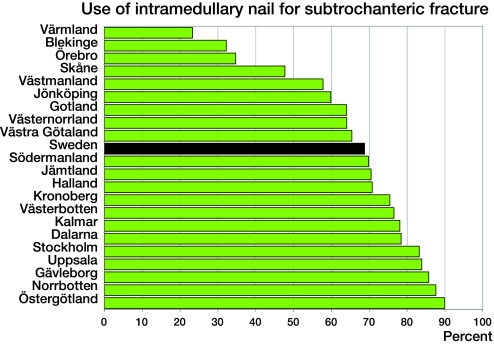
Use of intramedullary nail for subtrochanteric fracture (S72.2) in different Swedish counties, 2005–2007

For the 43,269 patients who were operated for hip fracture between January 1, 2005 and December 31, 2007, a comparison between the 21 counties in Sweden was made (Figures 5, 6, and 7). We found wide differences in the use of intramedullary nails for both pertrochanteric fractures (between 3% and 41%) and subtrochanteric fractures (between 23% and 90%). The use of arthroplasty for femoral neck fractures varied between 36% and 63%.

The geographic variations were notable also when comparing the re-admissions after 180 days. After femoral neck fracture, for example, the re-admission rate for any diagnosis varied between 19.5% and 31.0% and the re-admission rate due to hip complications varied between 3.8% and 10.8%.

## Discussion

In the best case, a change in treatment rationale is an evidence-based decision and in the worst case it can result from an urge to follow the trend. We have to rely mostly on a few published studies together with clinical experience summarized as “common expert opinion”, i.e. to some extent it is a subjective matter and perhaps even arbitrary.

Little is known in detail about the frequency of different surgical methods in a country. The hip fracture studies already published agree only on the point that there is a lack of agreement amongst surgeons on which method to use. Bhandari et al. (2005) performed an international survey and found that for displaced femoral neck fractures, surgeons preferred internal fixation for younger patients and arthroplasty for elderly ones. For patients between 60 and 80 years of age, there was no consensus as to the optimum treatment. In England, a telephone interview survey in 2000 showed that for active patients with a displaced femoral neck fracture, internal fixation, bipolar hemiarthroplasty, and unipolar hemiarthroplasty were roughly equally common as a first-hand choice (Crossman et al. 2002). A Norwegian survey found that one-third of the hospitals treated displaced femoral neck fractures with hemiarthroplasty and the rest used internal fixation with screws (Figved et al. 2005).

For extracapsular fractures, the sliding hip screw is still the gold standard according to evidence-based guidelines (Parker and Handoll 2008). The theoretical mechanical advantages of intramedullary nails (reduction of the distance between the implant and the joint, leading to less bending moment) has not yet been proven in clinical studies (Parker and Handoll, 2008). There might be specific types of fractures that are best served by use of an intramedullary nail or a biaxial sliding hip screw and plate, but this is still without sufficient support from scientific data. Even so, like Anglen and Weinstein (2008), we found an increase in the use of intramedullary nails for extracapsular fracture. The latter authors found by analysis of the database from the American Board of Orthopaedic Surgery that the rate of use of intramedullary nails went from 3% in 1999 to 67% in 2006, at the expense of the sliding hip screw. If no particular benefits are gained by using an intramedullary nail, cost effectiveness must be considered: the intramedullary nail may cost 3 to 6 times as much as a standard sliding hip screw. Our finding that patients with intramedullary nail had more re-admissions due to hip complications than those treated with sliding hip screw may have been biased by the possibility that intramedullary nails were more commonly used in comminuted fractures with a higher risk of complications. However, another study based on registry data found the same as we did: Aros et al. (2008) found a higher rate of revision surgery for pertrochanteric fractures treated with intramedullary nails and advised against their routine use in pertrochanteric fractures.

Variations in treatment of femoral neck fractures within a province have been reported from Canada (Jaglal et al. 1997, Cree et al. 2002). THA was used more often in hospitals associated with a medical school. During the study period (1981–1992), the use of hemiarthroplasty in Ontario increased from 45% to 61% (Jaglal et al. 1997).

Access to national health data registers, as in our study, provides real data for the whole country. The limitations are the lack of laterality in the register and in particular that the coding systems have too little detail. In the fracture groups, whether displacement or comminution is not explained by the diagnosis code.

The validity of national electronic databases has been questioned, for example by Lofthus et al. (2005). Their criticism of the Norwegian Patient Register points out the lack of the patient's personal identification number as a major source of error. The Swedish Patient Register, on the other hand, uses the unique 10-digit Swedish personal ID number, which allows tracing of re-admissions and reoperations. Continuous validation of health data registers is essential. For example, in 2008, co-processing was undertaken between the Swedish Patient Register and the Swedish Hip Arthroplasty Register (Kärrholm et al. 2008). The Hip Arthroplasty Register had a degree of coverage for total arthroplasties of 96%. The coverage in the Patient Register was lower (91%), to some extent explained by a generally low frequency of reporting from private hospitals to the register. We assume that the degree of coverage for hip fractures in the Swedish Patient Register might be actually better, as the few private hospitals in Sweden do not do emergency procedures.

The rapid change toward primary arthroplasty during the last decade is a new finding. Sernbo found that 2% and 10% of patients, respectively, were treated with arthroplasties in his nationwide surveys for 1990 and 1998 (Sernbo and Fredin 1993, Sernbo 1999). Sweden has been a stronghold of internal fixation for displaced femoral neck fractures after 3 promising studies in the 1980s (Strömqvist et al.1984, 1987, Rehnberg and Olerud 1989). Several randomized controlled studies that started in the 1990s, comparing internal fixation with arthroplasty, confirmed the superiority of the latter. These findings are summarized in meta-analyses (Bhandari et al. 2003, Parker and Gurusamy 2006, Rogmark and Johnell 2006). Thus, there is an evidence base for the observed change in treatment. Our finding that patients with internal fixation had more re-admissions than those treated with arthroplasty also supports the increasing use of arthroplasties.

For femoral neck fractures, the results are obscured by the fact that the diagnosis code does not discriminate between undisplaced and displaced fractures. Internal fixation is advocated as the treatment of choice for undisplaced fractures (Handoll and Parker 2008), which constitute one-third of the total (Thorngren and Hommel 2008); thus, two-thirds are displaced. Hence, the use of primary arthroplasty for only half of the fractures—as found in our study—suggests undertreatment.

Both our study and others highlight the obvious differences in treatment between hospitals, counties, and countries regarding hip fractures. Evidence-based guidelines are obviously needed to ensure good, consistent, and cost-effective care, and such algorithms are evolving in some centers, as published in USA and Australia (Shah et al. 2002, Chilov et al. 2003). The shift towards arthroplasties for displaced femoral neck fractures in Sweden must be seen as a response to several Swedish and international RCTs, resulting in a new treatment rationale. Hopefully a similar effort will be made during the next decade to provide a better evidence base to solve the treatment issues for trochanteric fractures.
